# Metformin partially reverses the carboplatin-resistance in NSCLC by inhibiting glucose metabolism

**DOI:** 10.18632/oncotarget.20663

**Published:** 2017-09-06

**Authors:** Yong Liu, Chunxi He, Xianping Huang

**Affiliations:** ^1^ Department of Cardiothoracic Surgery, The Second Affiliated Hospital and Yuying Children’s Hospital of Wenzhou Medical University, Wenzhou, Zhejiang, 325027, China

**Keywords:** NSCLC, carboplatin resistance, metformin, PKM2, glucose metabolism

## Abstract

Platinum-based chemotherapeutic drugs are irreplaceable for the treatment of advanced non-small cell lung cancer (NSCLC). However, acquired drug resistance has become a major obstacle for the clinical application of chemotherapy on NSCLC. In the present study, we established carboplatin-resistant NSCLC models on A549 and PC9 cell lines, which were named A549/R and PC9/R. Besides the low sensitivity of A549/R and PC9/R to carboplatin treatment, they exhibited higher metabolism rate of glucose compared to their parental A549 and PC9 cells, respectively. Mechanically, we confirmed that overexpression of PKM2 in A549/R and PC9/R was responsible for the high glucose metabolism and carboplatin resistance. Metformin, an antidiabetic drug, was observed to increase the sensitivity of carboplatin-resistant NSCLC cells to carboplatin treatment *in vitro* and *in vivo*. Mechanically, metformin decreased expression of PKM2 and subsequently inhibited the glucose uptake, lactate generation and ATP production in A549/R and PC9/R. Therefore, metformin promoted carboplatin-induced apoptosis through the mitochondria pathway. In addition, we demonstrated that metformin treatment also impaired the cross-resistance of A549/R and PC9/R to cisplatin, etoposide and 5-fluorouracil.

## INTRODUCTION

Due to the high potential of metastasis and low sensitivity to chemotherapy and/or radiation therapy, non-small cell lung cancer (NSCLC) is the leading cause of cancer-related deaths worldwide [1, 2]. For NSCLC patients without mutation of epidermal growth factor receptor (EGFR), chemotherapy is an irreplaceable treatment [3, 4]. Unfortunately, repeated use of chemotherapeutic drugs usually induces multiple drug resistance in cancers, especially in NSCLC [5]. It is urgent to explore and reveal the mechanisms by which chemoresistance occurs in NSCLC.

Platinum-based chemotherapy is considered as the first-line treatment for patients with advanced NSCLC. Carboplatin or cisplatin activates apoptosis signaling pathways of tumor cells by formatting inter- and intrastrand cross-links with DNA [6, 7]. However, due to the repeated use of platinum-based chemotherapeutic drugs, NSCLC cells develop strategies to resist apoptosis pathways [8]. Thus, adjuvant therapies are regard as indispensable treatments to reverse or delay the occurrence of drug-resistance.

Metformin (1,1-dimethylbiguanide hydrochloride) is a well-known oral medicine for treatment of type-II diabetes. Recent research demonstrates that the use of metformin in patients with diabetes may reduce the occurrence of cancers [9]. Furthermore, metformin treatment is reported to prevent lung adenoma formation and induce growth inhibition and cell cycle arrest in renal cancer cells [10, 11]. For adjuvant therapy of cancer, studies have indicated that metformin may reverse the resistance of breast cancer cells to doxorubicin by reducing the drug efflux [12]. Combination with metformin promotes sorafenib to suppress the proliferation and induce autophagy of hepatocellular carcinoma cells [13]. In lung cancer, metformin was found to reverse resistance to tyrosine kinase inhibitors and ALK inhibitors [14, 15]. It is indeed that metformin acts as a potential anti-cancer drug. The aim of this study is to investigate the role of metformin in reversing the carboplatin-resistance in NSCLC and explore the underlying mechanisms.

## RESULTS

### Overexpression of PKM2 is associated with carboplatin-resistance in NSCLC cells

**Figure 1:**
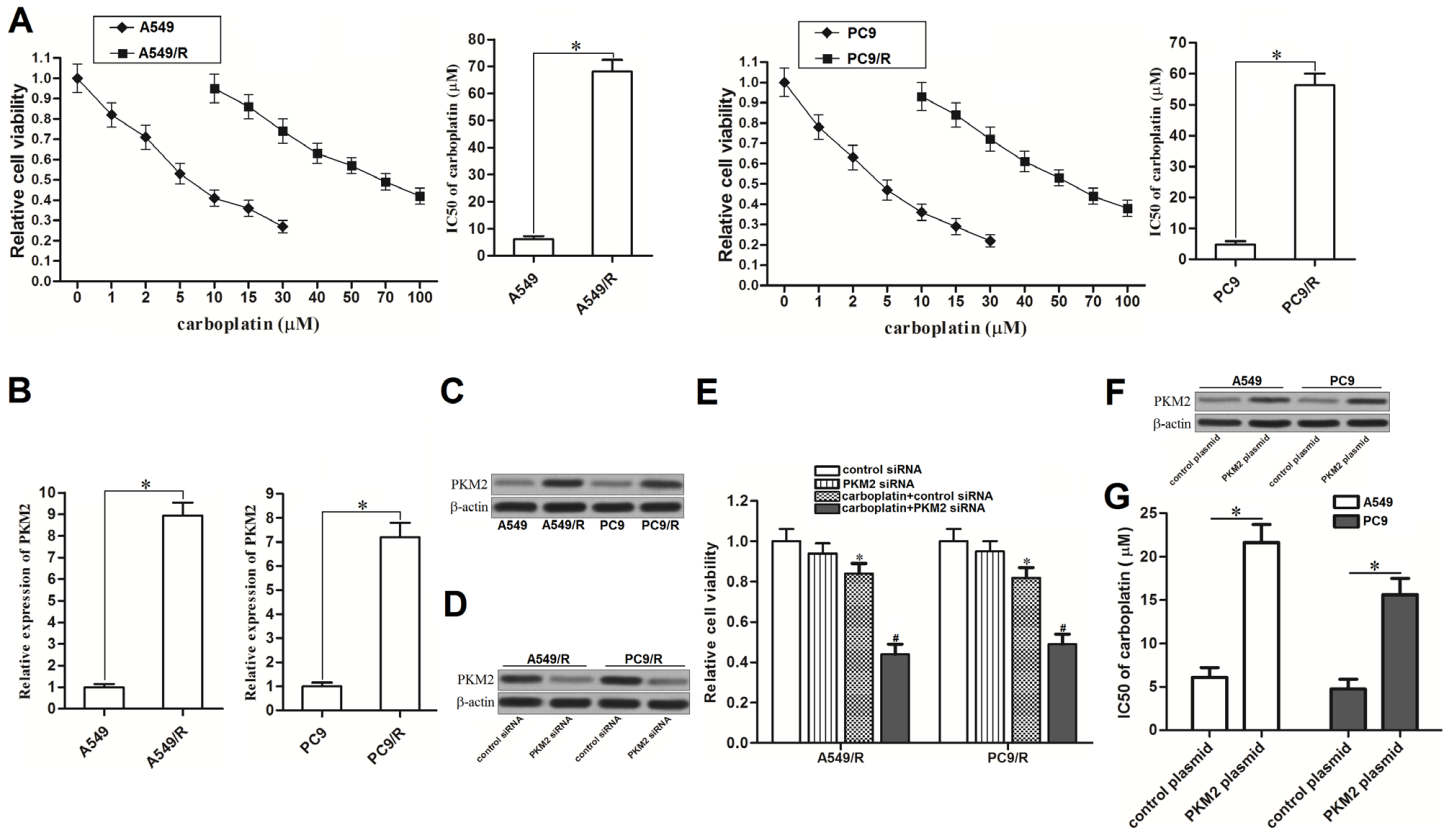
Overexpression of PKM2 induced resistance to carboplatin in NSCLC **(A)** After treatment with different concentrations of carboplatin for 48 h, cell viability of A549, A549/R, PC9 and PC9/R was measured by MTT assays. IC50 of carboplatin was determined according to the cell viability curve. *P<0.05. **(B)** qRT-PCR analysis was performed to detect the relative expression of PKM2 at mRNA level in A549, A549/R, PC9 and PC9/R. *P<0.05. **(C)** Western blot analysis was performed to detect the expression of PKM2 at protein level in A549, A549/R, PC9 and PC9/R. **(D)** After transfection with PKM2 siRNA (50 pmol/ml) for 24 h, expression of PKM2 in A549/R and PC9/R was measured by western blot analysis. **(E)** After transfection with PKM2 siRNA (50 pmol/ml) for 24 h, A549/R and PC9/R cells were treated with carboplatin (20 μM) for additional 48 h. Relative cell viability of them was detected by MTT assays. *P<0.05 *vs.* control siRNA group. ^#^*P*<0.05 *vs.* carboplatin + control siRNA group. **(F)** After transfection with PKM2 plasmid (2 μg/ml) for 24 h, expression of PKM2 in A549 and PC9 was measured by western blot analysis. **(G)** IC50 of carboplatin to A549 and PC9 was determined according to the cell viability curve. *P<0.05.

To study the chemoresistance in NSCLC, we established carboplatin-resistant NSCLC models on A549 and PC9 cell lines. As shown in Figure 1A, A549/R and PC9/R cells exhibited significant resistance to carboplatin compared to the corresponding A549 and PC9 cells, respectively. Interestingly, results of qRT-PCR showed that the expression of PKM2 was obviously overexpressed when the A549 and PC9 NSCLC cells became resistant to carboplatin (Figure 1B and 1C). It suggested that overexpression of PKM2 was associated with carboplatin-resistance in A549/R and PC9/R. To confirm this speculation, we transfected these A549/R and PC9/R cells with PKM2 siRNA. After transfection, expression of PKM2 was knockdown by its specific siRNA (Figure 1D). We then observed that knockdown of PKM2 significantly resensitized the A549/R and PC9/R to carboplatin treatment (Figure 1E). In addition, we transfected the routine A549 and PC9 cells with PKM2 plasmid (Figure 1F) before MTT assays. The results showed that enforced expression of PKM2 significantly reduced the sensitivity of routine A549 and PC9 cells to carboplatin treatment (Figure 1G). Taken together, we demonstrated that high level of PKM2 induced resistance to carboplatin in NSCLC.

### Metformin treatment decreases the expression of PKM2 in carboplatin-resistant NSCLC cells

**Figure 2:**
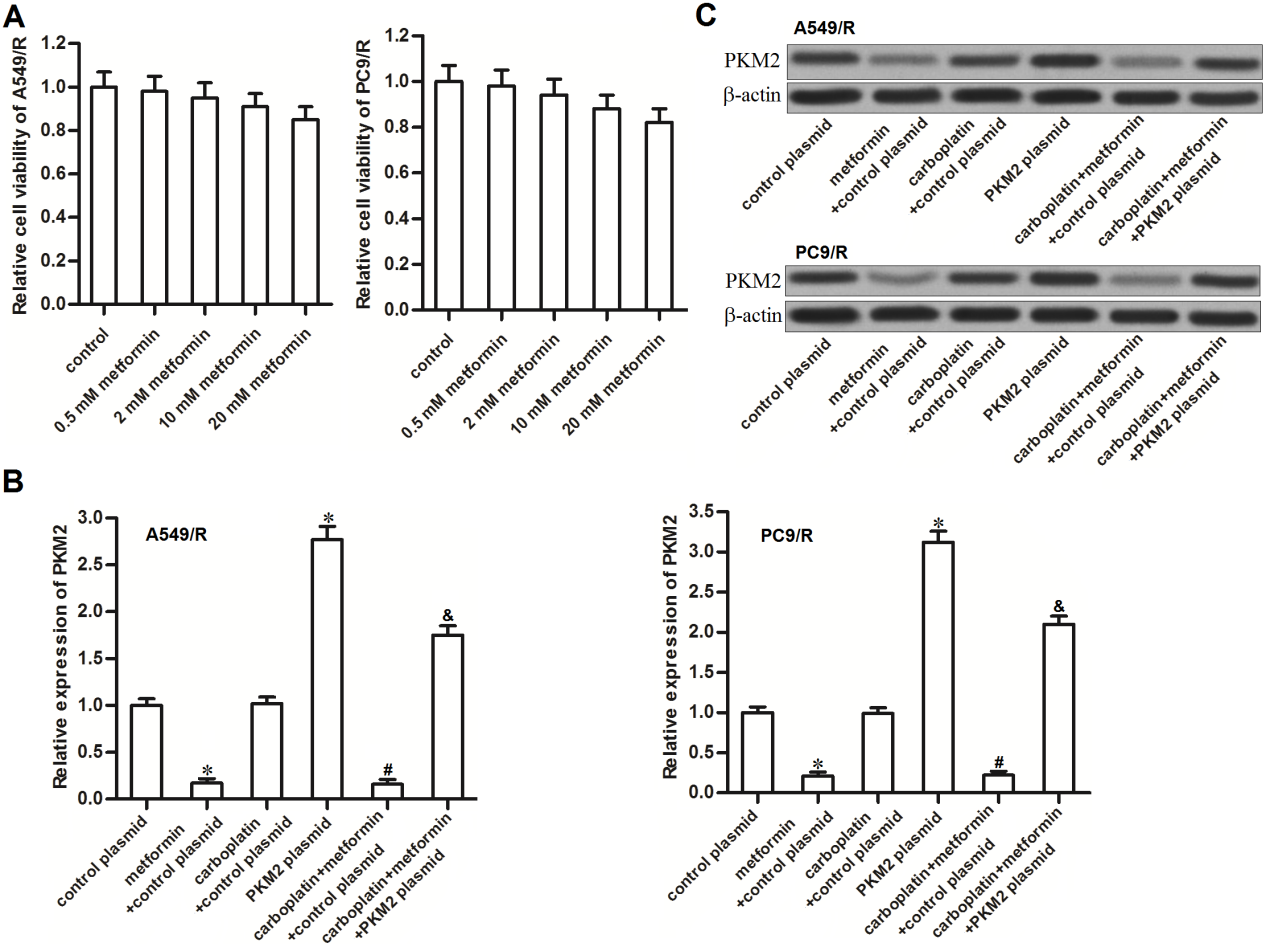
Metformin decreased the expression of PKM2 in A549/R and PC9/R **(A)** A549/R and PC9/R cells were treated with different concentrations of metformin. MTT assay was performed to detect the cell viability. **(B)** After treatment with metformin (2 mM), carboplatin (20 μM) and PKM2 plasmid (2 μg/ml), qRT-PCR analysis was performed to detect the relative expression of PKM2 at mRNA level in A549/R and PC9/R. *P<0.05 *vs.* control plasmid group. ^#^*P*<0.05 *vs.* carboplatin + control plasmid group. ^&^*P*<0.05 *vs.* carboplatin + metformin +control plasmid group. **(C)** Western blot analysis was performed to detect the expression of PKM2 at protein level in A549/R and PC9/R.

We first tested the cytotoxicity of metformin to carboplatin-resistant NSCLC cells. As shown in Figure 2A, cytotoxicity of metformin to A549/R and PC9/R was slight, even if the concentration of it was very high. We therefore chose a relative low concentration of metformin (2 mM) for co-treatment with carboplatin. Interestingly, we found that metformin (2 mM) but not the carboplatin (20 μM) decreased the expression of PKM2 in A549/R and PC9/R cells at mRNA level (Figure 2B) and protein level (Figure 2C). It suggested that metformin may play the subsidiary role in carboplatin-base chemotherapy by targeting PKM2. In addition, we observed that transfection with PKM2 plasmid significantly abolished the effect of metformin on decreasing the expression of PKM2.

### Metformin hampered the glucose metabolism of carboplatin-resistant NSCLC cells

**Figure 3:**
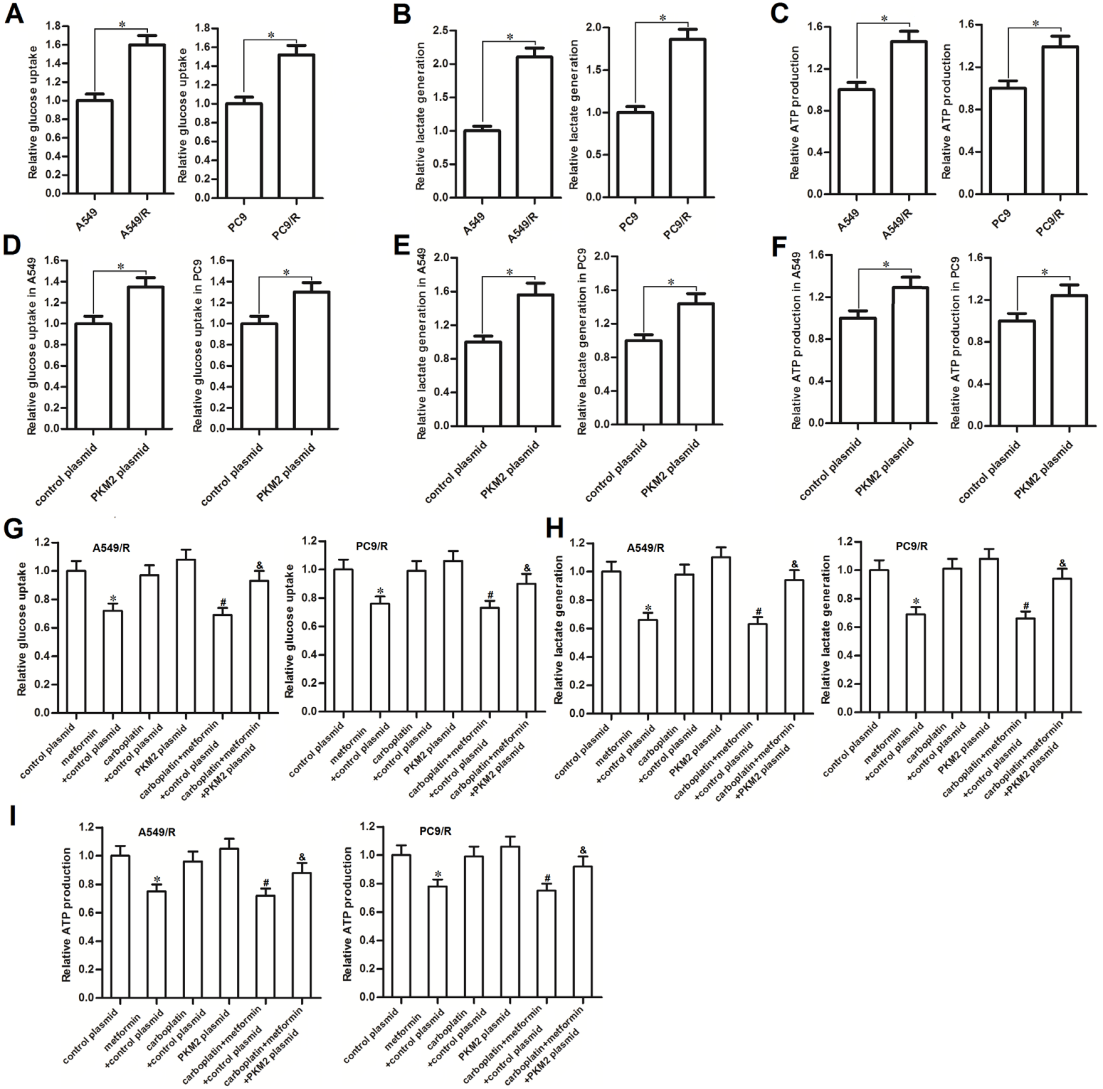
Metformin hampered the glucose metabolism of carboplatin-resistant NSCLC cells **(A)** Relative glucose uptake in A549, A549/R, PC9 and PC9/R cells. *P<0.05. **(B)** Relative lactate production in A549, A549/R, PC9 and PC9/R cells. *P<0.05. **(C)** Relative ATP production in A549, A549/R, PC9 and PC9/R cells. *P<0.05. **(D)** Relative glucose uptake in PKM2 plasmid (or control plasmid) transfected A549 and PC9 cells. *P<0.05. **(E)** Relative lactate production inPKM2 plasmid (or control plasmid) transfected A549 and PC9 cells. *P<0.05. **(F)** Relative ATP production in PKM2 plasmid (or control plasmid) transfected A549 and PC9 cells. *P<0.05. **(G)** Effect of metformin (2 mM), carboplatin (20 μM) and PKM2 plasmid (2 μg/ml) on changing the glucose uptake in A549/R and PC9/R cells. *P<0.05 *vs.* control plasmid group. ^#^*P*<0.05 *vs.* carboplatin + control plasmid group. ^&^*P*<0.05 *vs.* carboplatin + metformin +control plasmid group. **(H)** Effect of metformin (2 mM), carboplatin (20 μM) and PKM2 plasmid (2 μg/ml) on changing the lactate production in A549/R and PC9/R cells. *P<0.05 *vs.* control plasmid group. ^#^*P*<0.05 *vs.* carboplatin + control plasmid group. ^&^*P*<0.05 *vs.* carboplatin + metformin +control plasmid group. **(I)** Effect of metformin (2 mM), carboplatin (20 μM) and PKM2 plasmid (2 μg/ml) on changing the ATP production in A549/R and PC9/R cells. *P<0.05 *vs.* control plasmid group. ^#^*P*<0.05 *vs.* carboplatin + control plasmid group. ^&^*P*<0.05 *vs.* carboplatin + metformin +control plasmid group.

Recent research suggested that high rate of glucose metabolism facilitated cancer cells’ survival and contributed to chemoresistance [16]. In our study, we observed that glucose uptake was significantly increased when the NSCLC cells became carboplatin-resistance (Figure 3A). Furthermore, A549/R and PC9/R cells produced more amount of lactate and ATP compared with the A549 and PC9 cells, respectively (Figure 3B and 3C). Thus, we indicated that carboplatin-resistant NSCLC cells exhibited higher rate of glucose metabolism compared to their corresponding routine NSCLC cells. In addition, transfection with PKM2 plasmid into routine A549 and PC9 cells significantly increased the glucose uptake (Figure 3D), lactate generation (Figure 3E) and ATP production (Figure 3F). These results indicated that overexpression of PKM2 induced high rate of glucose metabolism in NSCLC. We next investigated the effect of metformin on carboplatin-resistance of NSCLC. After treatment with metformin and carboplatin in A549/R and PC9/R cells, we found that metformin but not the carboplatin significantly decreased glucose uptake and lactate production in both of these cells (Figure 3G and 3H). As the results, metformin strongly reduced the production of ATP in A549/R and PC9/R (Figure 3I). In addition, transfection with PKM2 plasmid significantly abolished the effect of metformin on reducing the glucose uptake, lactate production and ATP production. We therefore demonstrated that metformin hampered the glucose metabolism of carboplatin-resistant NSCLC cells by decreasing the expression of PKM2.

### Metformin sensitizes carboplatin-resistant NSCLC cells to carboplatin by decreasing the expression of PKM2 *in vitro*

**Figure 4:**
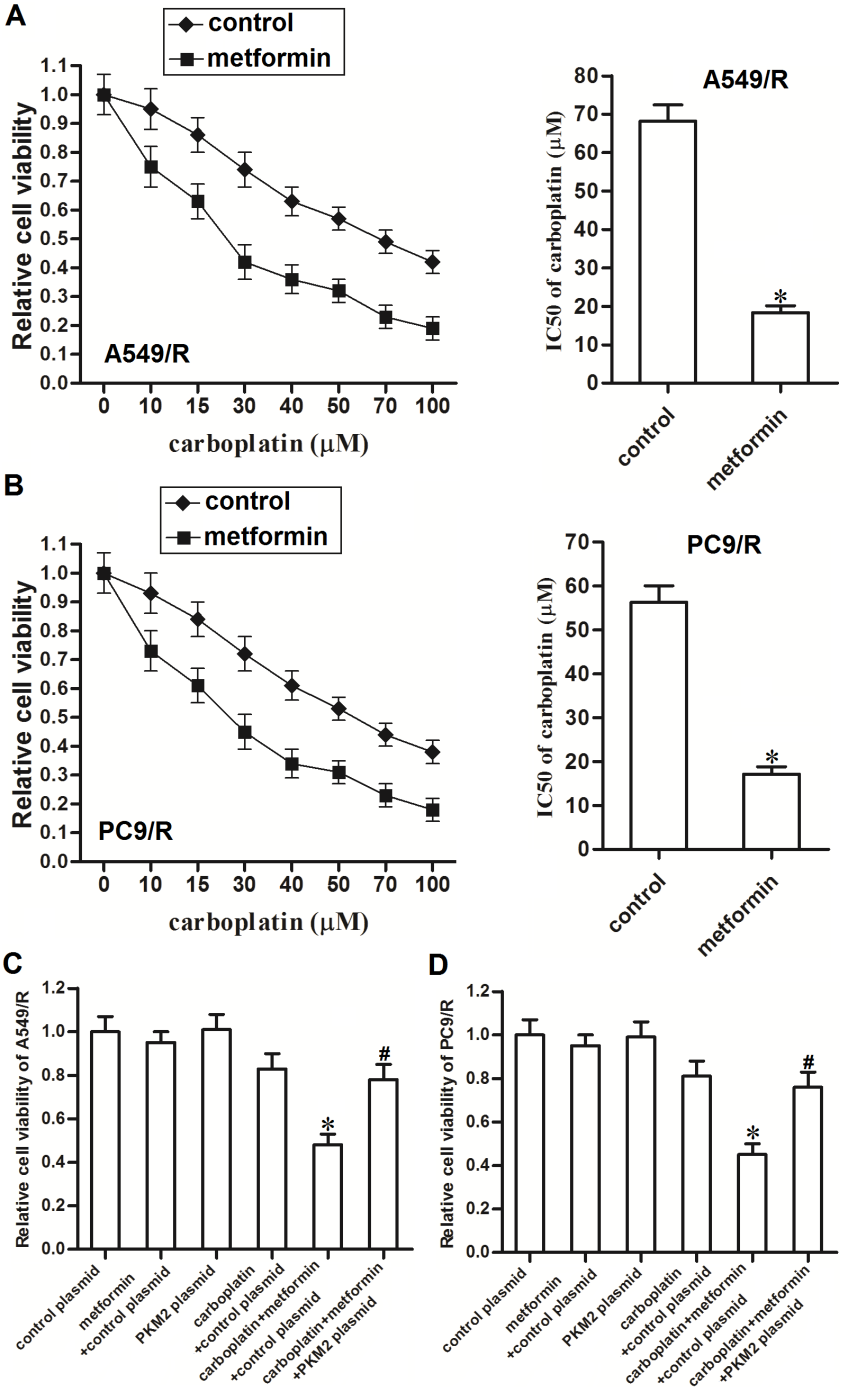
Metformin sensitizes A549/R and PC9/R cells to carboplatin by decreasing the expression of PKM2 **(A)** Metformin (2 mM) enhanced carboplatin-induced cell death in A549/R. *P<0.05 *vs.* control group. **(B)** Metformin (2 mM) enhanced carboplatin-induced cell death in PC9/R. *P<0.05 *vs.* control group. **(C)** PKM2 plasmid (2 μg/ml) abolished the promotion of metformin on carboplatin-induced (20 μM) cell death in A549/R. *P<0.05 *vs.* carboplatin + control plasmid group. ^#^*P*<0.05 *vs.* carboplatin + metformin + control plasmid group. **(D)** PKM2 plasmid (2 μg/ml) abolished the promotion of metformin on carboplatin-induced (20 μM) cell death in PC9/R. *P<0.05 *vs.* carboplatin + control plasmid group. ^#^*P*<0.05 *vs.* carboplatin + metformin + control plasmid group.

Results of MTT assays showed that metformin treatment significantly enhanced the carboplatin-induced cell death in A549/R (Figure 4A) and PC9/R (Figure 4B). We observed obvious decrease of carboplatin IC50 to A549/R and PC9/R mediated by metformin adjuvant therapy. However, we found that transfection with PKM2 plasmid significantly abolished the promotion of metformin on killing the A549/R (Figure 4C) and PC9/R (Figure 4D). Thus, we demonstrated that metformin was an effective adjuvant therapeutic drug for carboplatin-based chemotherapy in carboplatin-resistant NSCLC cells.

### Metformin sensitizes carboplatin-resistant NSCLC cells to carboplatin *in vivo*

**Figure 5:**
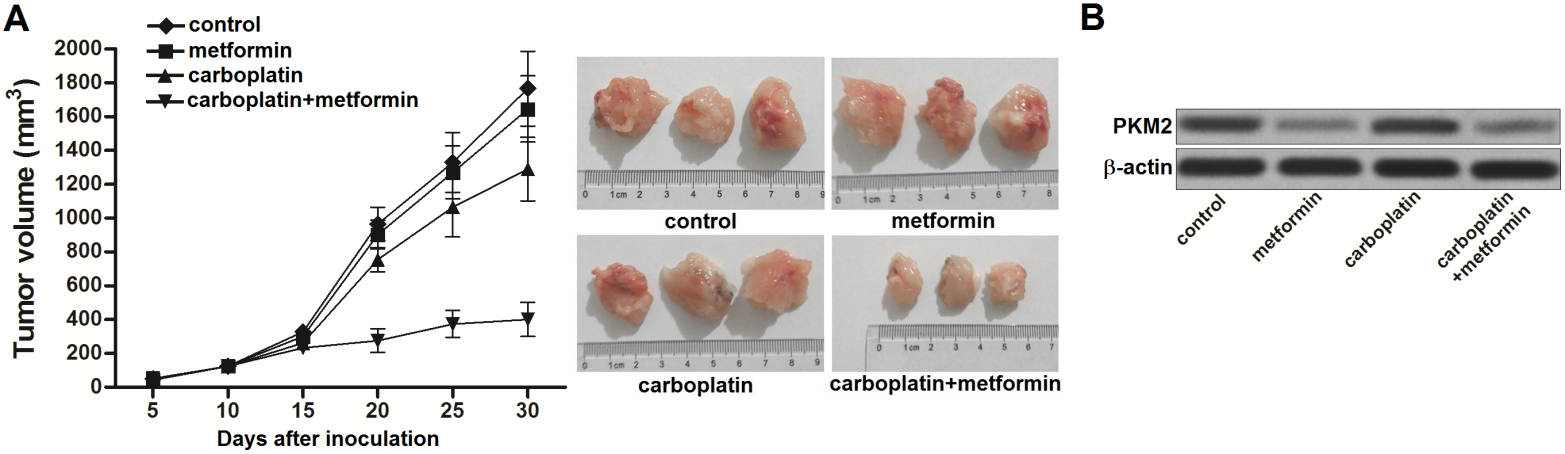
Metformin sensitizes carboplatin-resistant NSCLC cells to carboplatin *in vivo* **(A)** Anti-tumor effect of carboplatin and metformin on carboplatin-resistant NSCLC model. **(B)** Western blot assay was performed to analyze the expression of PKM2 at the protein level in carboplatin-resistant NSCLC tumor tissues treated with carboplatin and metformin.

To investigate the role of metformin in carboplatin-resistance in NSCLC, we established the *in vivo* model of NSCLC by using A549/R cells. We observed that single administration with carboplatin showed weak treatment effect on NSCLC. In contrast, although metformin single treatment didn’t reduce the tumor volume obviously, it strongly enhanced the anti-tumor effect of carboplatin on carboplatin-resistant NSCLC model (Figure 5A). After analyzing the expression of PKM2 in NSCLC tumor tissues by using western blot, we found that metformin significant decreased the expression of PKM2 in A549/R tumor model (Figure 5B). These results suggested that metformin can reverse the carboplatin resistance by inhibiting the expression of PKM2.

### Metformin promotes carboplatin-induced apoptosis through the mitochondria pathway in carboplatin-resistant NSCLC cells

**Figure 6:**
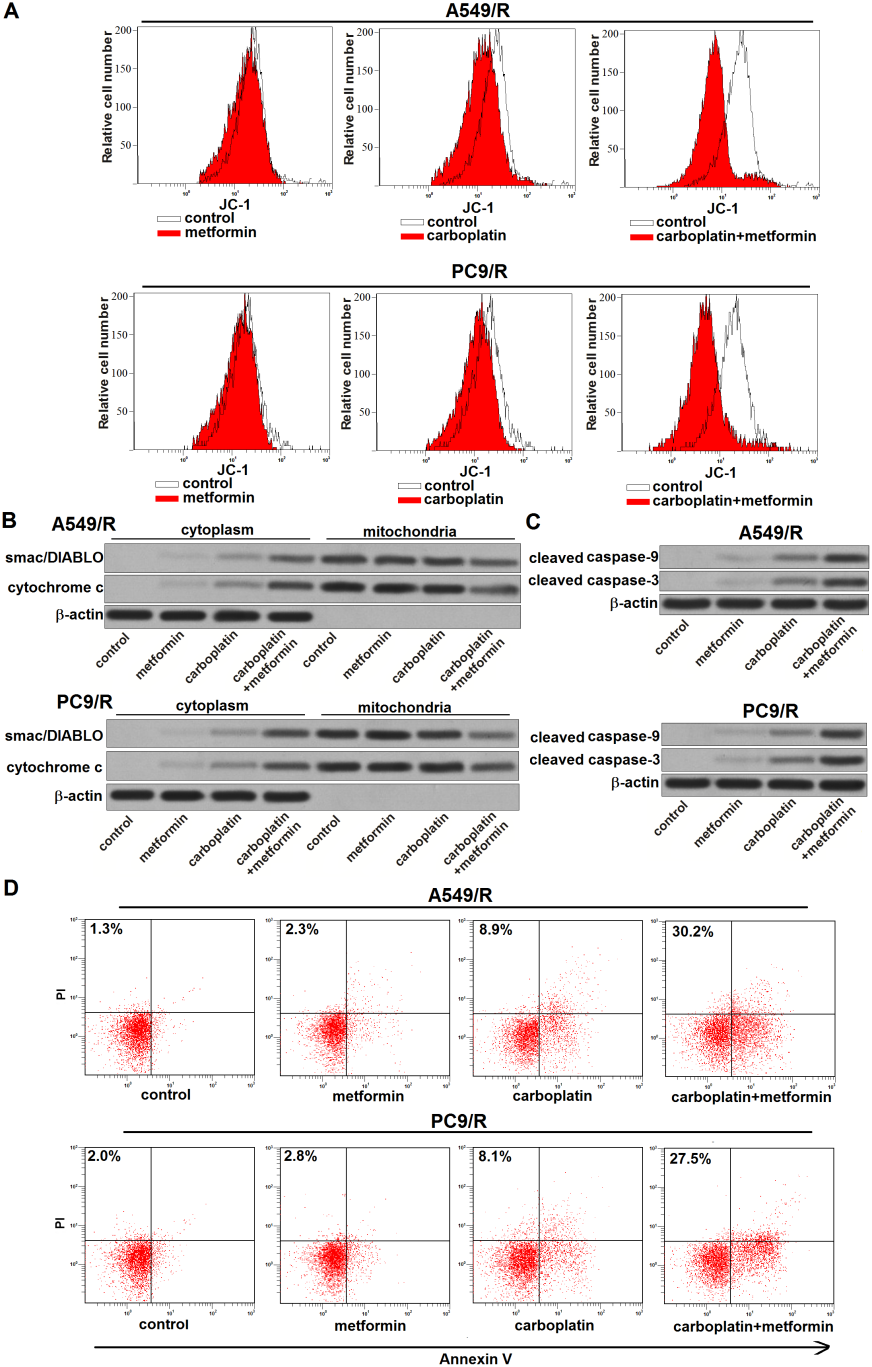
Metformin promotes carboplatin-induced apoptosis through the mitochondria pathway **(A)** After treatment with metformin (2 mM) and carboplatin (20 μM) for 48 h, mitochondrial membrane potential (MMP) of A549/R and PC9/R was detected by JC-1 staining on flow cytometry. **(B)** After treatment with metformin (2 mM) and carboplatin (20 μM) for 48 h, expression of smac/DIABLO and cytochrome c in cytoplasm or mitochondria was detected by western blot analysis. **(C)** After treatment with metformin (2 mM) and carboplatin (20 μM) for 48 h, cleavage of caspase-9 and caspase-3 in A549/R and PC9/R was evaluated by western blot analysis. **(D)** After treatment with metformin (2 mM) and carboplatin (20 μM) for 48 h, apoptotic rate of A549/R and PC9/R was measured by Annexin V and propidium iodide (PI) staining on flow cytometry.

Results of flow cytometry analysis showed that metformin significantly enhanced the effect of carboplatin on reducing the mitochondrial membrane potential (MMP) of A549/R and PC9/R (Figure 6A). Due to the damage of mitochondria induced by metformin and carboplatin co-treatment, mitochondria-derived pro-apoptotic inducers such as smac/DIABLO [17] and cytochrome c [18] were released from mitochondria into cytoplasm in A549/R and PC9/R (Figure 6B). As the results, caspase-9 and caspase-3 in A549/R and PC9/R were activated by these apoptotic inducers (Figure 6C). And finally, apoptosis was occurred in A549/R and PC9/R (Figure 6D). Taken together, these results demonstrated that adjuvant treatment with metformin could reverse the resistance of NSCLC cells to carboplatin-induced apoptosis through the mitochondria pathway.

### Metformin reverses cross-resistance of A549/R and PC9/R to cisplatin, etoposide and 5-fluorouracil

**Figure 7:**
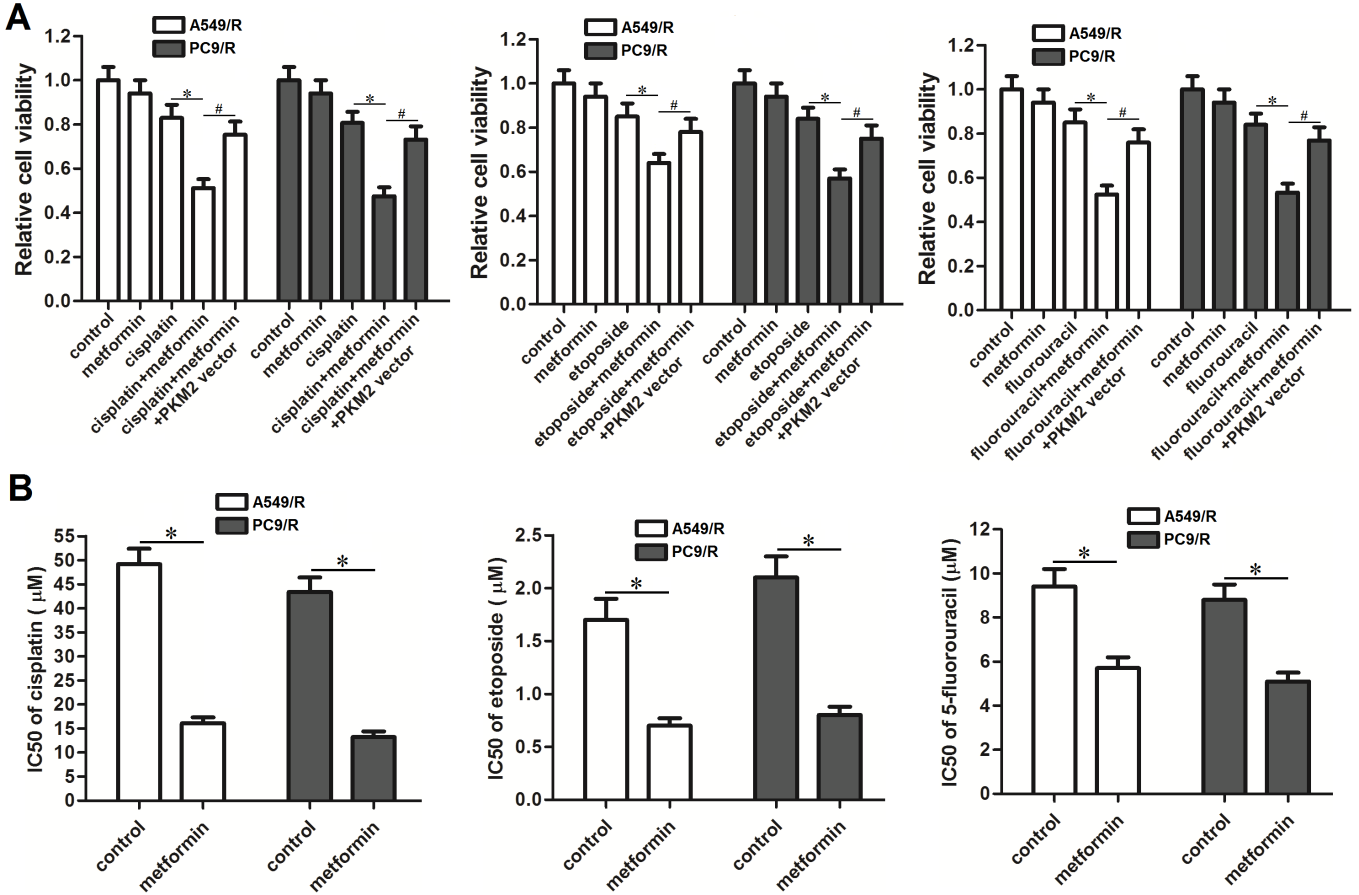
Effect of metformin on reversing the cross-resistance of A549/R and PC9/R **(A)** A549/R and PC9/R cells were treated with metformin (2 mM), cisplatin (15 μM), etoposide (0.5 μM) and 5-fluorouracil (5 μM) for 48 h. MTT assays were performed to detect the relative cell viability of A549/R and PC9/R cells. *P<0.05, ^#^*P*<0.05. **(B)** IC50 of cisplatin, etoposide and 5-fluorouracil to A549/R and PC9/R cells was evaluated according to cell viability curve determined by results of MTT assays. *P<0.05.

To investigate the role of metformin in multidrug resistance in carboplatin-resistant NSCLC cells, we treated the A549/R and PC9/R cells with metformin combined with cisplatin, etoposide or 5-fluorouracil. The results of MTT showed that metformin co-treatment significantly increased the sensitivity of A549/R and PC9/R cells to cisplatin, etoposide or 5-fluorouracil (Figure 7A). We found that adjuvant therapy with metformin obviously reduced the IC50 of cisplatin, etoposide and 5-fluorouracil to A549/R and PC9/R cells (Figure 7B). We demonstrated that that adjuvant treatment with metformin could reverse the cross-resistance of NSCLC cells.

## DISCUSSION

Carboplatin is one representative platinum-based chemotherapeutic drug. It exhibits effective activity against various solid tumors, especially NSCLC [19, 20]. Survival time of NSCLC patients is usually linked to the degree of clinical response to carboplatin treatment [21, 22]. However, NSCLC patients usually suffered from the drug resistance due to the repeated use of carboplatin [23, 24]. Mechanism by which carboplatin induces cell death of tumors is dependent on the DNA damage which is the strong apoptotic signaling [25, 26]. Thus, the carboplatin-resistance of tumor cells is exhibited as low response to apoptotic signaling caused by carboplatin treatment. In the present study, our established carboplatin-resistant NSCLC cells showed resistance to carboplatin treatment with significant low response to apoptosis pathway. Therefore, increasing sensitivity of carboplatin-resistant NSCLC cells to apoptotic signaling may represent an effective strategy against the chemoresistance.

Pyruvate kinase (PK) is a key enzyme of glycolysis. The Pyruvate kinase isoenzyme M1 (PKM1) is expressed in normal tissues while the PKM2 is strongly overexpressed in cancers. In glucose metabolism process, PKM2 converts phosphoenolpyruvate and ADP to pyruvate and ATP. Cellular pyruvate produces more amount of ATP through oxidative mitochondrial metabolism (OXPHOS). Indeed, the level of glucose metabolism and ATP is dependent on the level of PKM2 in cancer [27, 28]. Moreover, recent studies indicate that overexpression of PKM2 induces chemoresistance [29, 30]. In the present study, we observed significantly higher expression level of PKM2 in carboplatin-resistant NSCLC cells compared to routine NSCLC cells. Furthermore, knockdown of PKM2 in carboplatin-resistant NSCLC cells was found to sensitize these cells to carboplatin treatment. We proved the role of PKM2 in inducing the chemoresistance.

Due to the Warburg effect, cancer cells uptake higher level of glucose and require higher energy supply compare with the normal cells [31, 32]. Previous studies have indicated that cancer cells are sensitive to change of glucose metabolism, particularly the change of intracellular ATP level. It is reported that depletion of glucose metabolism and intracellular ATP induce the depression of drug efflux system, and sensitize cells to apoptosis pathway [33–37]. Thus, high level of glucose metabolism and intracellular ATP facilitate the occurrence of chemoresistance of cancer cells.

Metformin has been reported to inhibit the cell growth and reverse the drug-resistance in some cancers by reducing the production of ATP [38, 39]. In addition, although metformin has been reported to increase chemosensitivity and target PKM2 in routine cancer cells [40, 41], effect of metformin on cancer models with acquired drug resistance is unclear. In the present study, we demonstrated that metformin treatment decreased the expression of PKM2 in carboplatin-resistant NSCLC cells. PKM2 was shown as the target of metformin in these cells. Due to the decrease of PKM2, metformin was found to inhibit the glucose metabolism and reduce the ATP level in carboplatin-resistant NSCLC cells. As the results, metformin resensitized carboplatin-resistant NSCLC cells to carboplatin-induced apoptosis through the mitochondrial pathway. Generally speaking, these data explore the effect of metformin on reversing the acquired drug resistance of lung cancer. It is possible that high level of ATP and PKM2 making these resistant NSCLC cells more sensitive to metformin.

In conclusion, we provide reliable evidence that metformin treatment can partially reverse the resistance of NSCLC cells to carboplatin-induced apoptosis by inhibiting the glucose metabolism and ATP production. Furthermore, our data also indicated that the cross-resistance of NSCLC cells may be partially reversed by metformin. These findings suggest the value of metformin adjuvant treatment on platinum-based chemotherapy in NSCLC. However, further efforts should be made to explore the effect of metformin on other types of cancer and the biological mechanism by which metformin suppresses the expression of PKM2.

## MATERIALS AND METHODS

### Cell lines

Human NSCLC cell lines A549 and PC9 were purchased from American Type Culture Collection (ATCC, Rockville, MD, USA). Cells were cultured in DMEM medium supplemented with 10% fetal bovine serum (FBS, Gibco, Invitrogen). To establish the carboplatin-resistant NSCLC models, we exposed the A549 and PC9 cells with gradually increasing concentrations of carboplatin. Briefly, A549 and PC9 cells were initially treated with 1 μM carboplatin for 3 months. Then, the carboplatin concentration was increased every 3 weeks by 0.2 μM up to a final concentration of 3 μM. The established carboplatin-resistant A549 and PC9 cells were name as A549/R and PC9/R, respectively.

### Gain (loss)-of-function of PKM2

For overexpression of PKM2, open reading frame of PKM2 gene was amplified by PCR and then linked to pcDNA3.1 eukaryotic expression plasmid (Invitrogen). For knockdown of PKM2, the specific PKM2 small interfering RNA (siRNA) was purchased from Genechem Co., Ltd. (Shanghai, China). To perform the gain (loss)-of-function experiments of PKM2, A549/R and PC9/R cells were transient transfected with 2 μg/ml PKM2 plasmid or 50 pmol/ml PKM2 siRNA using Lipofectamine 2000 (Invitrogen) according to the manufacturer’s instructions.

### Cell viability and IC50

MTT assay was performed to detect the viability of NSCLC cells. First, 5 × 10^3^ NSCLC cells were seeded on 96-well plates and cultured at 37 °C. After treatment with chemotherapeutic drugs and metformin, cells were treated with MTT for additional 4 h. Cell viability was evaluated according to the absorbance measured at 490 nm by using an ELISA microplate reader (Sunrise Microplate Reader, TECAN, Switzerland). Half maximal inhibitory concentration (IC50) of chemotherapeutic drugs to NSCLC cells was calculated according to the corresponding cell viability curve.

### Quantitative real-time polymerase chain reaction (qRT-PCR)

Total RNAs were isolated from A549, PC9, A549/R and PC9/R cells using Trizol^®^ reagent (Invitrogen, USA) according to the manufacturer’s instructions. Subsequently, cDNA of these cells was reverse transcribed using the M-MLV Reverse Transcriptase (Invitrogen) according to the manufacturer’s instructions. QRT-PCR was performed with ABI PRISM 7500 Sequence Detection System (Applied Biosystems, USA) using SYBR Premix Ex Taq (TaKaRa, Japan). β-actin was used as normalization control to determine the relative expression of PKM2.

### Western blot analysis

NSCLC cells were lysed in 1 × SDS loading buffer containing protease inhibitors. Total proteins in cell lysates were then separated by 10% sodium dodecyl sulfate-polyacrylamide gel electrophoresis (SDS-PAGE), and then transferred to polyvinylidene fluoride (PVDF) membranes (Millipore, Billerica, MA, USA). Subsequently, these membranes were incubated with specifc antibodies for PKM2, smac/DIABLO, cytochrome c, cleaved caspase-9, cleaved caspase-3 and β-actin (Cell Signaling, USA) overnight. After incubating with appropriate HRP-conjugated secondary antibodies, the protein bands were detected by using an enhanced chemiluminescent substrate (Thermo Fisher Scientific, Inc, USA).

### Glucose, lactate and ATP assays

After treatment with carboplatin (20 μM) and metformin (2 mM), A549/R and PC9/R cells were collected and washed with PBS for twice. Relative glucose uptake, lactate production and ATP production were detected by using Amplex Red Glucose/Glucose Oxidase assay kit (Molecular Probes, USA), Lactate Assay Kit (BioVision, USA) and ATP Colorimetric/Fluorometric Assay Kit (Biovision), respectively.

### Analysis of apoptosis and mitochondrial membrane potential (MMP)

After treatment with carboplatin (20 μM) and metformin (2 mM), A549/R and PC9/R cells were collected and washed with PBS for twice. Cell apoptosis and MMP of A549/R and PC9/R were measured by using the Annexin V-FITC apoptosis detection kit (Sigma Aldrich, USA) and JC-1 mitochondrial dye (Biovision) respectively according to the manufacturer’s instructions.

### Tumor growth in nude mice

Four-week-old female immunodefcient nude BALB/c mice were purchased from Shanghai Super-B&K Laboratory Animal Corp., Ltd. (Shanghai, China). For xenograft, mice were injected subcutaneously into the right armpit with 5×10^6^ A549/R cells. carboplatin (5 mg/kg) and metformin (200 mg/kg) were administrated by intraperitoneal injection twice a week. Tumor size was measured every five days. Tumor volume was calculated according to the following formula: volume (V) = 1/2 × length × width^2^. The animal care and experimental protocols were approved by the Animal Care Committee of The Second Affiliated Hospital & Yuying Children’s Hospital of Wenzhou Medical University.

### Statistical analysis

All the experiments were independently repeated at least 3 times. Data are represented as mean ± SD and analyzed by using SPSS 15.0. Non-paired t test was used to estimate the statistical differences between two groups. One-way analysis of varianve (ANOVA) was used to determine the differences between three or more groups. A value of *P*<0.05 was considered to indicate a statistically significant difference.
